# Therapeutic Effects of Essential Oils and Their Bioactive Compounds on Prostate Cancer Treatment

**DOI:** 10.3390/pharmaceutics16050583

**Published:** 2024-04-24

**Authors:** Leticia Santos Pimentel, Luciana Machado Bastos, Luiz Ricardo Goulart, Lígia Nunes de Morais Ribeiro

**Affiliations:** Laboratory of Nanobiotechnology Professor Luiz Ricardo Goulart Filho, Institute of Biotechnology, Federal University of Uberlândia, Campus Umuarama, Bloco 2E, Sala 248, Uberlândia 38405-302, MG, Brazil

**Keywords:** essential oil, prostate cancer, natural treatment, natural products

## Abstract

Since prostate cancer (PCa) relies on limited therapies, more effective alternatives are required. Essential oils (EOs) and their bioactive compounds are natural products that have many properties including anticancer activity. This review covers studies published between 2000 and 2023 and discusses the anti-prostate cancer mechanisms of the EOs from several plant species and their main bioactive compounds. It also provides a critical perspective regarding the challenges to be overcome until they reach the market. EOs from chamomile, cinnamon, *Citrus* species, turmeric, *Cymbopogon* species, ginger, lavender, *Mentha* species, rosemary, *Salvia* species, thyme and other species have been tested in different PCa cell lines and have shown excellent results, including the inhibition of cell growth and migration, the induction of apoptosis, modulation in the expression of apoptotic and anti-apoptotic genes and the suppression of angiogenesis. The most challenging aspects of EOs, which limit their clinical uses, are their highly lipophilic nature, physicochemical instability, photosensitivity, high volatility and composition variability. The processing of EO-based products in the pharmaceutical field may be an interesting alternative to circumvent EOs’ limitations, resulting in several benefits in their further clinical use. Identifying their bioactive compounds, therapeutic effects and chemical structures could open new perspectives for innovative developments in the field. Moreover, this could be helpful in obtaining versatile chemical synthesis routes and/or biotechnological drug production strategies, providing an accurate, safe and sustainable source of these bioactive compounds, while looking at their use as gold-standard therapy in the close future.

## 1. Introduction

### 1.1. Overview of Prostate Cancer

Cancer has been considered one of the most alarming diseases in the last few decades, worldwide. It is a term used to cover more than 200 multifactorial diseases characterized by the uncontrolled growth and invasion of abnormal cells leading to the formation of tumors in healthy tissues [1]. Prostate cancer (PCa) is the second most prevalent cancer in men across the world and the fourth overall [2]. Its risks factors include age, ethnicity, diet, genetic, environmental factors and others. The incidence rate for men under the age of 50 years is 1 in 350 men, but it increases to nearly 60% in men over the age of 65 years [3]. PCa is a highly heterogeneous disease whose subtypes remain poorly understood [4].

In the early stages, PCa’s primary treatment is based on radiation and surgery. However, up to 53% of these cases progress to recurrence. Recurrence or metastatic tumors are usually treated with androgen deprivation therapy (ADT) in the form of gonadotropin-releasing hormone (GnRH) and androgen receptor (AR) antagonists, so progression can be prevented and patient survival can be improved. However, in most cases, despite their initial response, the cells become therapy-resistant, progressing to castration-resistant PCa (CRPC) and metastatic CRPC (mCRPC), which lead to lethal disease [5]. CRPCs are characterized by the consecutive activation of AR signaling through the expression of AR variants, AR gene/enhancer amplification, AR mutations, the overexpression of AR coactivators, AR-independent pathways and other mechanisms [6,7].

**Figure 1:**
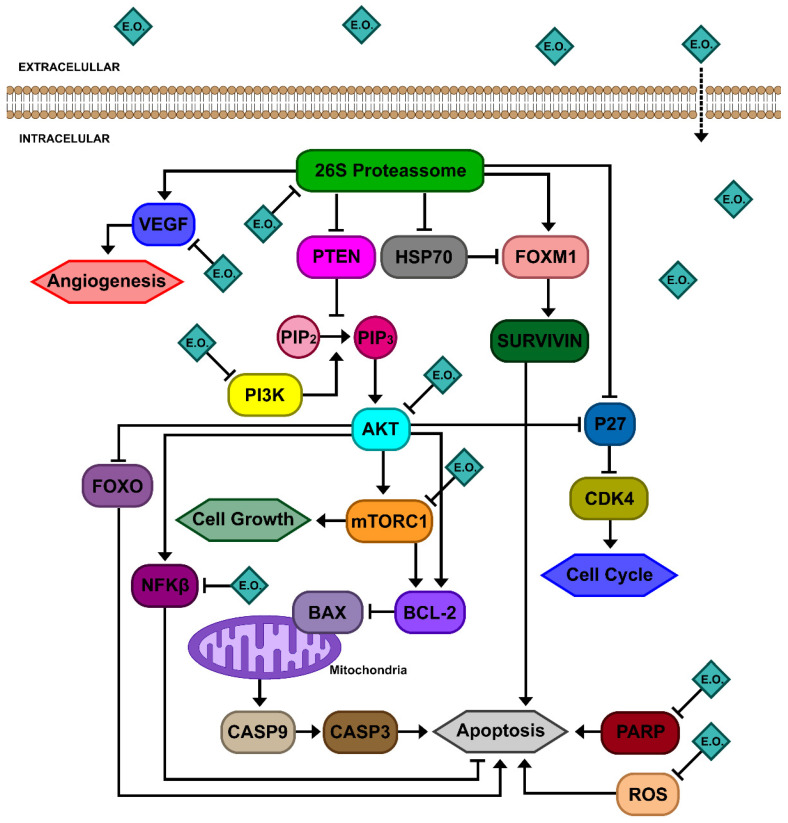
EOs and their bioactive compounds have roles in multiple pathways in PCa cells. EOs have cell membrane permeability and interact with several cellular targets involved in different pathways. EOs are able to interact with different targets of the PI3K/Akt pathway, leading to cell cycle arrest, apoptosis, the inhibition of cell growth and proliferation. EOs can also play a role as proteasome inhibitors, resulting in apoptosis, a decrease in cell proliferation and the suppression of angiogenesis. EOs modulate DNA damage repair mechanisms by acting as DNA polymerase inhibitors, which leads to PARP cleavage, resulting in apoptosis. EOs were demonstrated to induce mitochondrial stress, leading to changes in the expression of *BCL2/BAX* genes and membrane depolarization, resulting in the increased release of cytochrome-C to the cytoplasm and the induction of apoptosis. In addition, EOs increase intracellular ROS levels, resulting in PCa cells’ apoptosis.

In PCa cells, defective phosphatase and tensin homolog (PTEN) and the uncontrolled activation of phosphatidylinositol-3 kinase (PI3K)/AKT signaling frequently promotes cancer progression (Figure 1). The tumor suppressor PTEN is frequently mutated and shows a loss of function in PCa, allowing for the strong activation of the PI3K/AKT signaling pathway. Downstream of PI3K and AKT, the protein kinase mechanistic target of rapamycin (mTOR) plays an important role in cell growth regulation, and is often associated with tumorigenesis [8]. Other target molecules of downstream PI3K/AKT signaling, such as cyclin-dependent kinases (CDKs) and forkhead box subgroup O (FoxO), contribute to suppressing cell cycle control and apoptotic mechanisms, leading to PCa’s resistance to chemotherapeutic drugs [9].

The Ubiquitin-Proteasome (UPS) pathway plays a key role in the degradation of intracellular proteins involved in the regulation of several cellular processes, such as the cell cycle, DNA damage response, cell growth, apoptosis, angiogenesis and others (Figure 1). In PCa cells, the degradation of PTEN, cyclin-dependent kinase inhibitor p27 and Bcl2-associated x (Bax) by the UPS pathway are increased [10].

DNA damage repair (DDR) is another pathway altered in PCa cells. DDR deficiency induces cell dependence on poly (ADP-ribose) polymerase (PARP)-1 protein for DNA repair. The use of a PARP-1 inhibitor leads to PCa cell death [11,12].

Treatment options for PCa remain limited once chemotherapeutics present severe side effects, acting not only on cancer cells but also on normal tissues. Therefore, searching for new safe and efficient active compounds to treat PCa is necessary [13]. In this sense, natural products could be great potential therapeutics in cancer treatment, given that several pharmaceutical drugs have been developed from compounds derived from plants.

### 1.2. PCa Culture Cell Lines

Before proceeding with this review, it is important to understand the main PCa cell line models. DU145, PC3 and LNCaP were the first three PCa cell lines established from metastasized tumors and are the most frequently used to date, even with several other cell lines and sublines having been developed [14]. DU145 is an androgen-resistant cell line isolated in 1975 from the brain-metastatic PCa of a 69-year-old white man [15]. The androgen-resistant PC3 cell line was stablished in 1979 and derived from the lumbar-vertebral-metastatic PCa of a 62-year-old white man [16]. A subline was developed in 1984 from a PC3 xenograft tumor in an athymic mouse, called PC-3M, and revealed to be more aggressive than its parental PC3 cell line [17]. The androgen-responsive LNCaP cell line was established in 1980 and obtained from the needle aspiration biopsy of a lymph node metastatic lesion from a 50-year-old white man [18]. A subline from the vertebral metastasis of LNCaP xenografts in castrated mice was isolated in 1994 and called the C4-2 cell line. These cells became androgen-resistant upon interaction with bone fibroblasts [19]. Another subline (C4-2B) was developed from the bone metastasis of C4-2 xenografts in the same year [20]. The 22Rv1 is an androgen-responsive cell line isolated in 1999 from a CWR22R xenograft tumor in mice, which was obtainedfrom a patient with bone metastasis [21].

### 1.3. Essential Oils: Their Nature and Biological Activities

Essential oils (EOs) are natural lipid products present in various aromatic plants, derived from their secondary metabolites. They are usually obtained from leaves, flowers, fruits, seeds, buds, rhizomes, roots and/or bark [22,23], and are extracted by steam distillation and cold pressing processes [24]. EOs are defined as complex mixtures of approximately 20 to 60 components, with two or three of them found in high concentrations (20–70%) [25,26,27]. The chemical composition of their volatile fractions mainly includes mono- and sesquiterpenes, followed by several oxygenated derivatives, alcohols, aliphatic aldehydes and esters. On the other hand, non-volatile fractions comprise 1–10 wt% of EOs and are composed of carotenoids, fatty acids, flavonoids and waxes [26]. Generally, they are lipophilic, soluble in organic solvents and water-immiscible products [28].

Considering their wide chemical diversity, EOs have many properties and have been used since Ancient times as medicine. Among their activities, can be mentioned their antioxidant, anti-inflammatory, antibiotic, antiviral, antifungal, anti-parasitical, insecticidal, anticancer, wound healing, antihypertensive, analgesic and other clinical uses, such as a sedative, spasmolytic, analgesic, anesthetic or anxiolytic [29,30,31,32]. Furthermore, the global EO industry is valued at USD 18.6 billion and expected to reach USD 35.5 billion by 2028. This exponential growth can be attributed to increasing preference of people for a healthy lifestyle. The commercial use of EOs in aromatherapy, (mainly in spa and massage therapies) is their dominant application area, followed by the food and beverage industries [33]. Pharmaceutical fields also have a great interest in EOs’ properties, as they are rich sources of therapeutic compounds. 

EOs are considered promising candidates for cancer treatments through their antioxidant, antimutagenic, antiangiogenic, antiproliferation effects; enhancement of the immune system; enzyme induction; and modulation of multidrug resistance mechanisms [34,35]. In PCa cells (Figure 1), EOs were demonstrated to act on PI3K/AKT signaling and the Ubiquitin-Proteasome (UPS) pathway, resulting in apoptosis. Moreover, EOs are able to induce mitochondrial stress, leading to changes in the expression of Bcl-2/Bax genes and membrane depolarization, resulting in the increased release of cytochrome-C to the cytoplasm and the induction of apoptosis. In addition, EOs are able to increase intracellular reactive oxygen species (ROS) levels, resulting in the apoptosis of PCa cells [35]. All of these signaling pathways may be targets for novel therapeutic drugs for PCa treatments.

**Figure 2:**
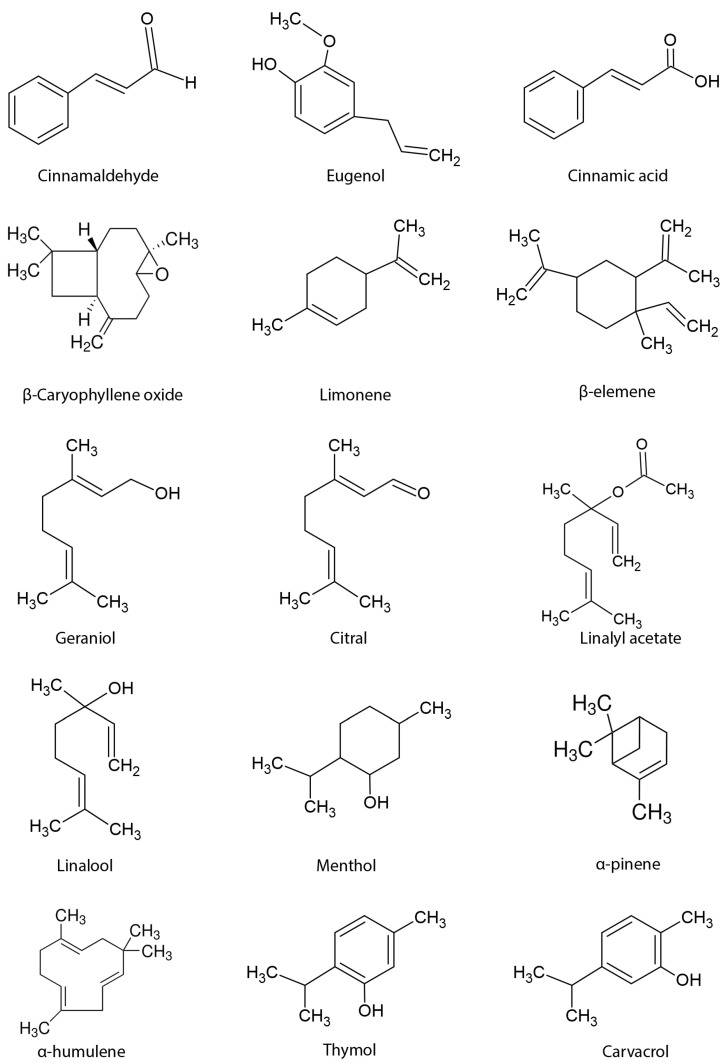
Representative illustration of the chemical structures of the bioactive compounds found in essential oils tested for anti-prostate cancer activity.

In this context, the present work carried out a review of the anticancer effects of EOs from several plant species, while also focusing on their main bioactive compounds as effective treatments against PCa cells. Table 1 lists the successful in vitro studies of EOs extracted from several plants against different PCa cell lines. Table 2 displays the in vitro results of the major EOs compounds. Figure 2 shows the chemical structures of the bioactive compounds found in EOs that have been tested for anti-prostate cancer activity. 

## 2. Methodology

This review follows the PRISMA (Preferred Reporting Items for Systematic Reviews and Meta-Analysis) verification protocol [101]. The keywords used in the search for reports were “Prostate cancer”, “Essential oil”, “Basil”, “Chamomile”, “*Citrus*”, “Turmeric”, “*Cymbopogon*”, “Ginger”, “Jasmine”, “Lavender”, “*Mentha*”, “Myrtle”, “Oregano”, “Rose”, “Rosemary”, “*Salvia*” and “Thyme”. The search was performed in the Pubmed, ScienceDirect, WebOfScience and Scopus databases, focusing on the anti-prostate cancer activity of EOs and their bioactive compounds.

The works that were considered eligible have met the following criteria for inclusion: (1) retrospective experimental and clinical studies in human and mammal models and/or cell lines, (2) research investigating the anti-prostate cancer activity of EOs, (3) works containing quantitative and qualitative data, (4) without any language restrictions, (5) published between 2000 and 2023 and (6) manuscripts published in journals with impact indexes. On the other hand, the works that were not considered eligible met the following criteria for exclusion: (1) not meeting the objectives of this article, (2) university theses, (3) book chapters or books and (4) review articles.

## 3. EOs and Their Bioactive Compounds Tested in PCa Cell Lines

### 3.1. Chamomile

Chamomile is one of the most common and well-known herbs widely used in medicinal applications and is from the Asteraceae family. It has been traditionally used to treat several diseases due to its antioxidant, anti-inflammatory, antibacterial, antifungal, antiparasitic, insecticidal, antidiabetic and anti-tumoral activities. Chamomile EO is obtained from flowers by steam distillation [102,103]. German chamomile (*Matricaria chamomilla*) and Roman chamomile (*Chamaemelum nobile*, also called *Anthemis nobilis*) EOs mainly contain the terpenoid α-bisabolol, and its oxides A and B, and chamazulene [103,104].

In a previous report, the cytotoxicity of chamomile (*Anthemis nobilis*) EO was evaluated in the PC3 PCa cell line and it was found to generate a dose-dependent decrease in the survival of cells with an IC_50_ value of 0.071% (*v*/*v*). At a concentration of 0.2% (*v*/*v*) the EO exhibited strong cytotoxicity, showing cell viability lower than 4% [38]. A comparative study has evaluated the viability of PC3, DU145 and LNCaP human PCa cells exposed to aqueous and methanolic extracts of chamomile (*Matricaria chamomilla*). The aqueous extract treatment resulted in a time- and dose-dependent reduction in cell viability for all the cell lines studied, with IC_50_ values ranging from 2000 to 3000 µg/mL after 72 h of treatment. Similar results were found in all the cells lines after treatment with the methanolic extract, with IC_50_ values ranging from 100 to 200 µg/mL after 24 h treatments. In addition, the exposure to both chamomile extracts for 48 h led to apoptosis in all PCa cell lines with the formation of internucleosomal DNA fragments. Still, such treatments did not reduce the viability nor induce apoptosis in virally transformed normal human prostate epithelial PZ-HPV-7 cells [105].

### 3.2. Cinnamon

The *Cinnamon* genus belongs to the Lauraceae family and includes more than 250 species of evergreen trees. It is a common spice that possesses several biological properties such as antioxidant, anti-inflammatory, antibacterial, antifungal, anti-parasitic, antidiabetic and antitumor characteristics [106,107]. Cinnamon (*Cinnamomum zeylanicum*, also called *Cinnamomum verum*) EO is obtained by steam distillation, usually from the bark, and its main components are cinnamaldehyde and eugenol [108,109].

The viability of PC3 PCa cells treated with cinnamon EO was determined by an MTT essay. The results revealed a dose-dependent decrease in the survival of cells with an IC_50_ value of 0.012% (*v*/*v*) [38]. Another work demonstrated that aqueous cinnamon extract and procyanidin B2 (PCB2), a hydrophilic component, induced a time- and dose-dependent decrease in the viability of PC3 and LNCaP cells. Moreover, apoptosis occurred through the inhibition of 26S proteasome activity, the induction of a dose-dependent increase in caspase-3 activity, a decrease in Akt protein levels and a decrease in anti-apoptotic (Survivin and XIAP) and angiogenic (VEGF and VEGF receptor) gene markers’ expression in both cancer cells lines [110]. An HPLC analysis of this aqueous cinnamon extract revealed the presence of high amounts of cinnamaldehyde [86].

Other relevant compounds that have been isolated from cinnamon include eugenol and cinnamic acid. Cinnamaldehyde, eugenol and cinnamic acid compounds were able to inhibit proteasome activity in both PC3 and LNCaP cells, resulting in an increase in the expression of the chaperone protein (Hsp70), which negatively regulates the expression of FoxM1, an oncogenic transcription factor. In addition, cinnamon compounds downregulated the expression of angiogenic factors (VEGF and VEGFR), acting as antiangiogenic agents. The treatment of tumor cells with cinnamon compounds also led to a dose-dependent decrease in cell viability with IC_50_ values of 14.3 μM for cinnamaldehyde, 73.2 μM for eugenol and 6.2 μM for cinnamic acid [86]. Eugenol also inhibits the growth of DU145 cells [90]. Other authors reported that cinnamaldehyde inhibited the growth of PCa-associated fibroblasts (CAFs) in a dose-dependent manner, showing an IC_50_ value of 74.66 µM. Prostate CAFs play an important role in promoting carcinogenesis and the progression of prostate cancer. In addition to that, cinnamaldehyde induced apoptosis via cell cycle arrest at the G_2_/M phase, increased ROS generation and decreased Glutathione (GSH) levels and caspase activation on prostate CAFs [87]. It also relieved the immunosuppressive effects on prostate CAFs in a Toll-Like Receptor 4 (TLR4)-dependent manner [88].

β-Caryophyllene oxide, a bicyclic sesquiterpene isolated from several EOs including cinnamon EO, inhibited the growth of PC3 PCa cells in a time- and dose-dependent manner. The sesquiterpene has also induced apoptosis by inhibiting PI3K/AKT/mTOR/S6K1 signaling, which reduced mitochondrial membrane potential and cytochrome c release, activating caspase-3, the cleavage of PARP, ROS generation, the downregulation of anti-apoptotic proteins (Bcl-2, Bcl-xL, survivin, IAP-1 and IAP-2), cell cycle protein cyclin D_1_ and the upregulation of the tumor suppressor protein p53 and CDK inhibitor p21. In addition, β-Caryophyllene oxide downregulated proteins linked with metastasis and angiogenesis (COX-2 and VEGF) [78,79]. Another report showed that β-Caryophyllene oxide inhibited the cell growth, invasion and constitutive STAT3 activation in DU145 cells. The constitutive activation of STAT3 is often active and linked with proliferation, survival, invasion, metastasis and angiogenesis in tumor cells [80].

### 3.3. Citrus Species

The genus *Citrus* (Rutaceae family) is one of the most ancient and popular crops [111]. Citrus fruit are a significant source of vitamins such as ascorbic acid. The biological activities of citrus fruit include antioxidant, anti-inflammatory, antibacterial, antifungal, antitumor activities and others. The use of their EOs as natural preservatives in the food industry has been extensively studied [112]. Generally, the EOs of citrus fruit are obtained via a cold pressing method from different parts of the plants, such as fruit peel and leaves, and are mainly composed by limonene [109,112,113,114,115].

Mandarin (*Citrus reticluata*)-EO-treated PC3 PCa cells exhibited a dose-dependent decrease in viability with an IC_50_ value of 10.97 µg/mL. In addition, mandarin EO induced apoptotic DNA fragmentation, ROS generation and a decrease the expression of B-cell lymphoma 2 (Bcl2) and Murine Double Minute 2 (MDM2) genes, while it increased the expression of the p53 and Bax genes [47]. Lemon (*Citrus limon*) and grapefruit (*Citrus paradisi*) EO treatments in the PC3 cell line revealed a dose-dependent decrease in the survival of cells with IC_50_ values of 0.083% and 0.094% (*v*/*v*), respectively [38]. A navel orange (*Citrus sinensis*) EO treatment revealed a time- and dose-dependent inhibition of the proliferation of 22RV1 cells by inducing apoptosis. Its IC_50_ values at 24, 48 and 72 h were 45.74, 42.83, and 39.79 µg/mL, respectively [50]. Kumquat (*Fortunella margarita*) EO showed time- and dose-dependent antiproliferative activity against LNCaP cells by inducing apoptosis and the inhibition of inflammation. The treatment increased the Bax/Bcl2 ratio and induced the cleavage of caspase-3, indicating induction of apoptosis. Additionally, it decreased expression of inflammatory transcription factor NFκB and Cox-2, a downstream product of NFκB, indicating its inhibition of inflammation [42].

Limonene, a hydrocarbon monoterpene present in several citrus oils, inhibited the growth of DU145 cells in a dose-dependent manner with an IC_50_ value of 2.8 nM. In combination with docetaxel, limonene has enhanced cell growth inhibition. Moreover, this combined treatment induced ROS generation and enhanced apoptotic cell death by inducing cytoplasmic histone-associated DNA fragmentation; caspase-3 and caspase-9 cleavage; the upregulation of p21, p53 and Bad; and the downregulation of Bcl-xL and cleavage PARP protein. No effect on caspase-8 cleavage or on the expression of Bax and Bcl-2 were observed [94].

### 3.4. Turmeric

The genus *Curcuma* from the Zingiberaceae family is constituted by perennial rhizomatous herbs and includes about 100 species [116]. Turmeric (*C. longa*) has been widely explored as a coloring and flavoring agent, as well as in the pharmaceutical industry due to its antioxidant, anti-inflammatory, antimicrobial, antiviral, antitumor, antidiabetic, antiasthmatic, hypoglycemic, neuro- and dermoprotective and other properties. Its EO is obtained from its rhizome and is mainly composed of α and β-turmerone [116,117].

Turmeric EOs obtained from *C. longa* rhizomes collected from 20 different habitats were investigated for their cytotoxicity activity against LNCaP cells. It was revealed that they led to an inhibition of cell growth with IC_50_ values ranging from 16.41 to 124.27 µg/mL [55]. Turmeric-EO-treated PC3 prostate cancer cells revealed an inhibition of cell growth with an IC_50_ value of 97.94 µg/mL [52].

β-elemene reduced PC3 and DU145 cell growth in a time- and dose-dependent manner, with IC_50_ values of 105, 102 and 96 µg/mL at 24, 48 and 72 h; and 75, 70 and 66 µg/mL at 24, 48 and 72 h, respectively. Treatment with β-elemene also induced apoptosis in a time- and dose-dependent manner through cleaved caspase-3 and caspase-9, increased PARP and Bcl-2 downregulation [81].

### 3.5. Cymbopogon Species

The *Cymbopogon* genus belongs to the Andropoganeae family and includes more than 144 species of aromatic grass plants comprising lemon grass (*C. citratus*), tsauri grass (*C. giganteus*), citronella (*C. nardus*) and camel grass (*C. schoenanthus*). *Cymbopogon* species have several biological activities such as antioxidant, anti-inflammatory, antibacterial, antifungal, insecticidal and antitumor activities among others. Cymbopogon EOs are extensively used in the fragrance, cosmetics, food and flavor industries. They are obtained by the hydrodistillation of the species’ leaves and their main components are geraniol, citral and citronellal, depending on the species [118]. 

Treatments with lemon grass and tsauri grass EOs in PC3 and LNCaP cells showed that they act as antiproliferative agents. The IC_50_ values of lemon grass were 32.1 µg/mL for PC3 cells and 6.34 µg/mL for LNCaP. Tsauri grass exhibited values around 303.2 µg/mL for PC3 cells and 160.1 µg/mL for LNCaP [46]. Citronella EO also has antiproliferative activity in LNCaP cells (IC_50_ values of 58 µg/mL), caused by cell cycle arrest at the G_2_/M phase and changes in cell morphology [39]. Camel grass EO showed antiproliferative activity in LNCaP cells (IC_50_ values of 135.53 µg/mL), which was associated with its anti-migration property and cell cycle arrest in the G2/M phase [119].

Geraniol, an acyclic monoterpene, inhibited the cell growth of PC3 in the range of 0.25–1 mM. In the cells treated with geraniol, increased LDH and caspase-3 activity were observed, as were induced mitochondrial membrane depolarization and cell cycle arrest at the G_1_ phase. Additionally, the expressions of four cyclin isotypes (cyclin A, B, D, and E), two CDK family members (CDK1 and CDK4) and two anti-apoptotic Bcl-2 family members (Bcl-2 and Bcl-w) were reduced while the expressions of two CDK inhibitory proteins (p21 and p27) and two pro-apoptotic Bcl-2 family members (Bax and BNIP3) were noticeably elevated in cells treated with geraniol. In addition, the geraniol treatment, in a PC3 cell xenograft tumor in nude mice, efficiently suppressed tumor growth by inducing apoptosis and cell cycle arrest [91]. Geraniol-treated PC3 cells also experienced induced autophagy, inhibited AKT/mTOR signaling [92] and downregulated E2F8 transcription factor [93].

Citral, an acyclic monoterpene, when used as a treatment in PC3 and PC3M PCa cells, inhibited cell viability in a dose-dependent manner with IC_50_ values of 10 and 12.5 µg/mL. Citral treatment also reduced the cells’ clonogenic potential, induced morphological alterations and the expulsion of lipid droplets by the activation of AMPK protein expression and the subsequent downregulation of AMPK pathway genes such as SREBP1, ACC and HMGR. In addition, the Citral treatment induced apoptosis via DNA fragmentation, the upregulation of Bax and the downregulation of Bcl-2 expression [89].

### 3.6. Ginger

Ginger (*Zingiber officinale*), a member of the Zingiberaceae family, is a common spice used as a flavoring agent in beverages and food preparations. Ginger is also known to possess many therapeutic uses such as antioxidant, anti-inflammatory, anti-microbial and antitumor activities [120,121]. The EO of ginger is obtained from its rhizome and contains mainly zingiberene, curcumene and farnesene molecules, in its complex mixture [121]. 

A ginger EO treatment in PC3 cells exhibited a dose-dependent cytotoxicity, showing an IC_50_ value of 0.077% (*v*/*v*) [38]. Another work also showed the time- and dose-dependent antiproliferative activity of ginger EO in PC3 and LNCaP cell lines, with IC_50_ values of 0.42 mg/mL and 0.38 mg/mL, respectively [37]. Moreover, the ginger extract treatment was able to inhibit the proliferation of PC3 (IC_50_ of 250 µg/mL), LNCaP (IC_50_ of 75 µg/mL), DU145 (IC_50_ of 95 µg/mL), C4-2 (IC_50_ of 512 µg/mL) and C4-2B (IC_50_ of 240 µg/mL) PCa cells, while no effect was observed in normal prostate epithelial cells (PrEC). In addition, a treatment with ginger extract led to cell cycle arrest in PC3 cells by decreasing cyclin D1, cyclin E and CDK4 levels and increasing p21 and the CDK4 inhibitor. Additionally, a ginger extract induced apoptosis in PC3 cells by increasing Bax and decreasing Bcl2 expressions, releasing cytochrome *c*, increasing caspase-3 activity and cleaving PARP protein levels. In LNCaP cells, ginger extract also led to cell cycle arrest and increased caspase-3 activity. Mice treated with ginger extract had their tumor tissue suppressed and no detectable toxicity in their normal tissues was reported [122].

### 3.7. Lavender

The genus *Lavandula* (Lamiaceae family) comprises herbs popularly known for relieving stress, anxiety and depression. It also has other important therapeutic properties, such as antioxidant, anti-inflammatory, antibacterial and antitumor activities [33,44,45]. Lavender EO is obtained from flower heads and foliage by steam distillation and its main components are linalyl acetate and linalool molecules [123,124].

The cytotoxicity activity of lavender (*L. stoechas*) EO was evaluated in PC3 PCa cells. A dose-dependent decrease in the survival of cells was found, with an IC_50_ value of 0.05% (*v*/*v*) [38]. *L. stoechas* EO was also cytotoxic in LNCaP PCa cells [43]. PC3 cells were also treated with lavender (*L. officialis*) EO and showed a slight reduction in the viability of the cells [45]. A lavender (*L. angustifolia*) EO treatment in PC3 and DU145 cells revealed a time-dependent decrease in the viability of cells, with IC_50_ values of 0.037% and 0.199% (*v*/*v*), respectively. A treatment with the major components, linalyl acetate and linalool, also showed a potent cytotoxicity against both cell lines, with IC_50_ values of 4.98 µM and 11.74 µM in PC3 and 3.06 µM and 7.22 µM in DU145 cells, respectively. In addition, EO, linalyl acetate and linalool treatments inhibited migration and induced apoptosis via cell cycle arrest at the G_2_/M phase in PC3 and at the S phase in DU145 PCa cell lines. Lavender EO and its components have also inhibited the tumor growth of human PCa xenografts in mice [44]. Moreover, a linalool treatment inhibited the cell proliferation of 22Rvl PCa cells in a dose-dependent manner, showing an IC_50_ value of 3.38 mM. Linalool has also induced apoptosis, cell cycle arrest at the G_0_/G_1_ phase and a significant increase in expression of Bax, Bcl-2, p53, TRAIL receptors 1 (DR4), TRAIL receptors 2 (DR5) and cleaved caspases. Treatment with linalool suppressed significantly the tumor growth of human PCa xenografts in mice by inhibiting tumor cell proliferation and apoptosis [95].

### 3.8. Mentha Species 

The genus *Mentha* belongs to the Lamiaceae family and possess more than 25 species including *M. arvensis* (wild mint), *M. piperita* (peppermint), *M. longifolia* (horsemint) and *M. spicata* (spearmint). The EOs and extracts from *Mentha* species have been used since ancient times for the treatment of several gastrointestinal system diseases. These EOs are obtained from the leaves and flowering aerial parts of these species and their main components are carvone, limonene, menthone and menthol [41,124,125].

The cytotoxicity of four *Mentha* species-based EOs has been assessed in LNCaP PCa cells. All of them showed strong cytotoxicity against the cancer cell line, with IC_50_ values of 55.7, 95.7, 52.0 and 90.0 µg/mL, respectively [41]. Another work has evaluated the cytotoxicity of spearmint EO in PC3 cells and it a dose-dependent decrease in the survival of cells was found, with an IC_50_ value of 0.088% (*v*/*v*) [38].

Menthol can evoke a cold sensation, mediated by cold-sensitive transient receptor potential melastatin 8 (TRPM8). This receptor was found to be expressed at high levels in several tumors, including PCa tissue and the PC3, LNCaP and DU145 culture cell lines [96]. Researchers have demonstrated a dose-dependent decrease in viability of PC3, LNCaP and DU145 cells treated with menthol [96,97,98,99]. Menthol also inhibits DU145 cell migration and induces cell cycle arrest at the G_0_/G_1_ phase [99]. On the other hand, the PC3 and LNCaP cell cycles were not affected by menthol treatments [97]. Although all three tested cell lines have expressed the TRPM8 receptor, menthol activity seems to not be mediated by the TRPM8 pathway [96,97].

### 3.9. Rosemary

Rosemary (*Rosmarinus officinalis*), a member of the Lamiaceae family, has been used as stimulant, analgesic and anti-inflammatory compound. Additionally, the pharmacological properties of rosemary include its antioxidant, antibacterial, antifungal, antiviral and antiproliferative activities. Its EO is mainly composed of eucalyptol, α-pinene and camphor [126].

A report has evaluated the cytotoxicity of rosemary EO in the LNCaP cell line and an IC_50_ value of 180.9 µg/mL has been found [53]. Rosemary extract treatments in LNCaP and 22Rv1 prostate cells inhibit cell growth (with IC_50_ values of 27 and 13.3 µg/mL, respectively), induced cell cycle arrest at the G_1_/G_2_ phase and G_2_ phase, respectively, and induced apoptosis by increasing the expression of Bax and cleaved caspase-3 [127]. Rosemary extract also inhibited cell growth, survival and migration, inducing apoptosis in PC3 PCa cells, while it had no significant effect on the proliferation of PNT1A normal PCa cells [128].

α-pinene, one of the most common terpenes of rosemary EO, possesses a strong inhibitory effect on the growth of PC3 and DU145 PCa cells, with IC_50_ values of 2.9 and 5.8 µM, respectively. In addition, α-pinene induced apoptosis and cell cycle arrest at the S phase in PC3 cells and at the G_2_/M phase in DU145. In xenograft tumors, α-pinene suppresses, significantly, their growth and induces apoptotic cells death [77].

### 3.10. Salvia Species

The *Salvia* genus (Lamiaceae family) comprises about 1000 species that are herbaceous, suffruticosus or shrubby perennial plants. Sage EO is commonly used as an analgesic, anti-inflammatory, anti-viral, or antitumor compound, in the treatment of cardiovascular and liver diseases, and in food and cosmetics industries, among other applications. Sage EO is obtained via a steam distillation process and its main components are linalyl acetate and linalool [129,130].

A group of researchers studied the effects of *S. aurea*, *S. Judaica* and *S. viscosa* EOs on DU145 PCs cells. Treatment with the three EOs in the range of 12.5–50 µg/mL induced a dose-dependent decrease in cell growth. However, only at 50 µg/mL was an increase in lactate dehydrogenase (LDH) observed, indicating the induction of necrosis, cell death, at this concentration. Furthermore, at 12.5 and 25 µg/mL, the EOs treatments caused an increase in DNA fragmentation, caspase activity and the Bax/Bcl-2 ratio and, at all three concentrations, it caused an increase in ROS generation and a decrease in GSH levels in a dose-dependent manner [54]. Although an *S. officialis* EO treatment in the range of 5–400 µg/mL in LNCaP cells has no significant effect, treatment with the sesquiterpene α-humulene showed high cytotoxicity with an IC_50_ value of 11.24 µg/mL [76].

### 3.11. Thyme

The *Thymus* genus of the Lamiaceae family contains about 400 species of perennial aromatic, evergreen or semi-evergreen herbaceous plants [131,132]. *Thymus* species have been used to treat several cardiorespiratory and gastrointestinal diseases due to their antioxidant, antibacterial, antifungal, antiviral and antispasmodic, among other, properties. Moreover, thyme EO is used in food and cosmetics as an antioxidant and preservative [133]. It is a product obtained from the flowering tops of *Thymus vulgaris* or *Thymus zygis,* or both species, by steam distillation. The main components found in thyme EO are thymol and carvacrol [131,134,135]. 

A previous work has evaluated the cytotoxicity of Thyme (*T. vulgaris*) EO in the PC3 cell line and found a dose-dependent decrease in the survival of cells with an IC_50_ value of 0.01% (*v*/*v*). At a concentration of 0.2% (*v*/*v*), the EOexhibited strong toxicity, showing a cell viability lower than 4%. Compared to the other nine EOs evaluated in this study, thyme EO showed the strongest cytotoxicity [38].

Studies have reported that thymol, a monoterpene phenol, has a cytotoxic effect on the viability of PC3 and DU145 PCa cells. Thymol-treated cells, after 24, 48 and 72 h, showed time- and dose-dependent decreases in cancer cell growth, exhibiting IC_50_ values of 711, 601 and 552 µM for PC3 and 799, 721 and 448 µM for DU145, respectively. Thymol has also induced apoptosis in both cell lines in a dose-dependent manner, but the mechanism of this action has not been elucidated yet. [100].

Carvacrol, a monoterpene phenol, was found to have antiproliferative and apoptotic action against human PCa DU145 cells in a time- and dose-dependent manner, showing IC_50_ values of 84.39 µM and 42.06 µM after 24 and 48 h of treatment, respectively. Carvacrol has also induced apoptosis via caspase-3 activation, an increase in ROS generation and cell cycle arrest at the G_0_/G_1_ phase [82]. In PC3 cells, carvacrol revealed similar effects, inhibiting proliferation, migration and invasion. Apoptosis acted in reducing cell viability with the induced activation of caspase-3, -8 and -9, induced high levels of ROS, disrupted the mitochondrial membrane potential, arrested the cell cycle at the G_0_/G_1_ phase, upregulated Bax and downregulated Bcl-2 expression. In addition, carvacrol decreased the expression of Notch1, Jagged-1, cyclin D1 and CDK4 and the increased expression of p21 [83].

Carvacrol also inhibits cell growth, migration and invasion in both PC3 and DU145 cell lines by decreasing the TRPM7-like current and reducing Matrix metalloproteinase-2 (MMP-2) protein expression and F-actin dynamics. In addition, both the PI3K/Akt and MEK/MAPK signaling pathways seems to be involved in the anticancer effects of carvacrol [84,85]. Trindade et al. successfully complexed carvacrol with β-cyclodextrin in order to enhance its solubility and anticancer activity. The resulting inclusion complex reduced the cell viability and migration of PC3 PCa cells in a dose-dependent manner [136].

### 3.12. Other Species

Jasmine (*Jasminum grandiflorum*)- and rose (*Rosa centifolia*)-EO-treated PC3 cells revealed a dose-dependent decrease in the survival of cells with IC_50_ values of 0.022% and 0.04% (*v*/*v*), respectively [38]. Oregano (*Origanum vulgare*) EO, which has a high content of carvacrol, cymene and linalool, was successfully encapsulated in a nanoemulsion and significantly inhibited the growth of PC3 PCa cells in a dose-dependent manner, with an IC_50_ value of 13.82 µg/mL. A treatment with an oregano-based nanoemulsion also induced apoptosis by causing cellular damage, DNA fragmentation, enhanced Bax expression, cytochrome c release, caspase-3 activation and decrease in Bcl2 expression [51]. Myrtle (*Myrtus communis*) EO induced a time- and dose-dependent decrease in the viability of PC3 and DU145 cells, while no effect was observed in normal PNT1A cells. In addition, myrtle EO showed antimigratory and proapoptotic properties [49].

Bayala et al. evaluated the cytotoxicity of several EOs towards PC3 and LNCaP cell lines. In addition to ginger, which has already been mentioned here, *Ocimum basilicum*, *Lippia multiflora* and *Ageratum conyzoides* EO treatments showed strong antiproliferative activity against both PCa cell lines. In PC3 cells, their IC_50_ values were 0.45, 0.30 and 0.49 mg/mL, respectively, and in LNCaP cells they were 0.46, 0.58 and 0.35 mg/mL, respectively. However, *Ocimum americanum*, *Hyptis spicigera* and *Eucalyptus camaldulensis* EOs have shown no antiproliferative effects [37]. *Hyptis suaveolens*-EO-treated LNCaP cells have showed a dose-dependent decrease in cell viability with an IC_50_ value of 163.01 µg/mL and cell cycle arrest at the G_0_/G_1_ phase [56].

*Zataria Multiflora* EO, which is mainly composed of carvacrol, terpinene, cymene and thymol, has inhibited the cell viability of PC3 PCa cells with an IC_50_ value of 26.3 µg/mL after 48 h of treatment. It also has induced apoptosis by increasing ROS generation, DNA fragmentation, cell cycle arrest at the G_0_/G_1_ phase, caspase activation, the upregulation of Bax and the downregulation of Bcl-2 expression. A combined treatment with this EO and doxorubicin improved the effects on PC3 cells in comparison to the pure drug [75].

*Hedychium spicatum* EO, mainly composed of β-pinene and eucalyptol, has inhibited the viability of PC3 cells in dose-dependent manner with an IC_50_ value of 21.88 µg/mL. It also induced apoptotic cell death, cell cycle arrest at the G2/M and S phases, intracellular ROS accumulation, mitochondria depolarization and increased caspase-3, -8, and -9 levels. In addition, an *H. spicatum* EO decreased Bcl-2 and Bcl-xL and increased Bax and Bak protein levels [66]. Other species, *Hedychium genus*, *H. coccineum*, *H. gardnerianum*, *H. greenii* and *H. griffithianum*, also showed antiproliferative activity against PC3 cells [67].

EOs obtained from the flowers and leaves of *Artemisia arborescens* have shown inhibitory growth activity against LNCaP and DU145 with IC_50_ values of 5.6–6.1 and 5.1–5.7 µg/mL, respectively. They have also induced DNA fragmentation and increased ROS levels [62].

An EO from *Xylopia frutescens* leaves, which is rich in E-caryophyllene and commonly known as embira, showed cytotoxic activity against PC-3M-metastatic PCa cells, with an IC_50_ value of 40 µg/mL [40]. An EO from the leaves of *Guatteria pogonopus*, which is mainly composed of γ-patchoulene, (E)-caryophyllene and β-pinene, showed similar results, with an IC_50_ value of 17.0 µg/mL [72].

*Pinus mugo* EO, which has a high content of β-caryophyllene, bornyl acetate and α-pinene, inhibited the viability of DU145 cells in time- and dose-dependent manner with an IC_50_ value less than 50 µg/mL. Their constitutive STAT3 activation signaling cascade was down-modulated, which decreased the expression of anti-proliferative as well as anti-apoptotic genes and proteins such as cyclin D1, Bcl-2, survivin, XIAP, Cox2 and IL-6. It also induced a quick decrease in GSH levels and an increase in ROS generation. In addition, apoptotic cell death was induced by caspase-3, and PARP cleavage induction and cell migration were inhibited in a dose-dependent manner [74].

*Abies balsamea* [36], *Symphyopappus itatiayensis*, *Myrciaria floribundus*, *Talauma ovata*, *Psidium cattleyanum*, *Nectandra megapotamica* [71], *Amomum tsao-ko* [59], *Solanum erianthum*, *Solanum macranthum* [48], *Achillea wilhelmsii* [57], *Annona sylvatica* [61], *Hypericum hircinum* [68], *Anaxagorea brevipes* [60], *Guatteria elliptica* [65], *Bursera glabrifolia* [63], *Iryanthera polyneura* [69], *Aloysia polystachya* [58], *Euodia ruticarpa* [64], *Perralderia coronopifolia* [73] and *Liquidambar orientalis* [70] EOs demonstrated antiproliferative activity in PC3 PCa cells. In contrast, Emani et al. evaluated a *Nepeta cataria* EO (15–500 µg/mL) treatment in PC3 and DU145 PCa cells and no significant effect was observed [137]. Clove (*Syzygium aromaticum*) EO, which has a high content of eugenol and β-caryophyllene, also showed no significant effect on the DU145 cell line in the range of 100–300 µL/mL [138]. The same was found for an *Anemopsis californica* EO treatment for PC3 cells [139].

## 4. Conclusions

PCa is the second most prevalent cancer in men worldwide and its treatment options remain limited. EOs are natural products that have been used in medicine since ancient times. Due to their wide chemical diversity, they possess several therapeutic properties, including anticancer activity. 

Several EOs have been tested for their anti-prostate cancer property, such as chamomile, cinnamon, *Citrus* species, turmeric, *Cymbopogon* species, ginger, lavender, *Mentha* species, rosemary, *Salvia* species, thyme and other species, and shown to inhibit cell growth and migration, induce apoptosis, modulate the expression of apoptotic and anti-apoptotic genes and suppress angiogenesis. The major therapeutic compounds of these EOs have also been tested for anti-prostate cancer properties and showed similar results.

EOs and their constituents may be promising candidates for cancer treatments since they have potent therapeutic effects and are biocompatible, abundant and cheaper than current chemotherapeutics. In addition, novel anticancer compounds should be investigated in EOs’ molecular structures to contribute to the search for novel bioactives for innovative pharmaceutical preparations for anti-prostate cancer therapies.

## 5. Perspectives

There are many works that provide relevant results regarding EOs effects as a candidate treatment for several diseases, but clinical tests are still scarce. As observed, for PCa, there are very few in vivo tests and no clinical trials. The most challenging aspects of EOs, which limit their clinical use, are their highly lipophilic nature, physicochemical instability, photosensitivity and high volatility. The processing of EO-based products in the pharmaceutical field may be a good alternative to circumvent EOs limitations, resulting in the benefit of their therapeutic properties. New technologies for EO-based drug delivery system, such as nanoencapsulation, should be considered to ensure its high bioavailability and, consequently, its therapeutic effects. Different nanosystems could be developed, such as nanoemulsions, liposomes, lipid nanoparticles (SLN/NLC), polymer nanocapsulesand cyclodextrin. Although they are different nanocarriers, all of them have the ability to upload hydrophobic molecules with success. Therefore, they can offer physical protection to EO together with their nanometric particle sizes, which improve EOs solubility and prevent their degradation, hydrolysis and evaporation, allowing their safe and efficient administration via different routes. 

However, more efforts are still necessary in order to develop stable, scaled-up, biocompatible and efficient EO-based nanosystems. Another challenge in the use of EOs in disease treatments is their composition variability, due to the difficulty of standardizing the composition of bioactive compounds whichcurrently limits their use to only as adjuvant therapy. Identifying the bioactive compound, its therapeutic effect and its chemical structure could open perspectives for novel research in this field. Chemical synthesis or biotechnological drug production strategies can provide accurate, safe and sustainable sources of these bioactive, allowing for their use in gold-standard therapies. 

We hope that this review can stimulate further research in this field in order to provide promised PCa treatments in the close future.

## Figures and Tables

**Table 1:** Essential oils investigated for in vitro anti-prostate cancer potential, and the major results reported.

Common Name	Scientific Name	Cell Line	Effects	References
Balsam fir	*Abies balsamea*	PC3	Inhibition of cell growth	[36]
Basil	*Ocimum basilicum*	PC3 and LNCaP	Inhibition of cell growth	[37]
Chamomile	*Anthemis nobilis*	PC3	Inhibition of cell growth and induction of apoptosis	[38]
Cinnamon	*Cinnamomum zeylanicum*	PC3	Inhibition of cell growth	[38]
Citronella	*Cymbopogon nardus*	LNCaP	Inhibition of cell growth, cell cycle arrest and changes to cell morphology	[39]
Embira	*Xylopia frutescens*	PC-3M	Inhibition of cell growth	[40]
Ginger	*Zingiber officiale*	PC3	Inhibition of cell growth	[38]
		PC3 and LNCaP	Inhibition of cell growth	[37]
Grapefruit	*Citrus paradisi*	PC3	Inhibition of cell growth	[38]
Horsemint	*Mentha* *longifolia*	LNCaP	Inhibition of cell growth	[41]
Jasmine	*Jasminum grandiflora*	PC3	Inhibition of cell growth	[38]
Kumquat	*Fortunella margarita*	LNCaP	Inhibition of cell growth, induction of apoptosis, increased Bax/Bcl2 ratio, induced caspase-3 cleavage and inhibition of inflammation due to decreased expression of NFκB and Cox-2	[42]
Lavender	*Lavandula stoechas*	PC3	Inhibition of cell growth	[38]
		LNCaP	Inhibition of cell growth	[43]
	*Lavandula angustifolia*	PC3 and DU145	Inhibition of cell growth and migration, induction of apoptosis and cell cycle arrest	[44]
	*Lavandula officialis*	PC3	Slight Inhibition of cell growth	[45]
Lemon	*Citrus limon*	PC3	Inhibition of cell growth	[38]
Lemon grass	*Cymbopogon citratus*	PC3 and LNCaP	Inhibition of cell growth	[46]
Lippia	*Lippia multiflora*	PC3 and LNCaP	Inhibition of cell growth	[37]
Mandarin	*Citrus reticluata*	PC3	Inhibition of cell growth, induction of apoptotic DNA fragmentation and ROS generation; alterations in the expression of apoptotic and anti-apoptotic genes	[47]
Mentrasto	*Ageratum conyzoides*	PC3 and LNCaP	Inhibition of cell growth	[37]
Mullein nightshade	*Salanum erianthum*	PC3	Inhibition of cell growth	[48]
Myrtle	*Myrtus communis*	PC3 and DU145	Inhibition of cell growth and migration and induction of apoptosis	[49]
Navel orange	*Citrus sinensis*	22RV1	Inhibition of cell growth and induction of apoptosis	[50]
Oregano	*Origanum vulgare*	PC3	Inhibition of cell growth; induction of apoptosis; cellular damage; DNA fragmentation; enhanced Bax expression, cytochrome c release and caspase-3 activation; and decreased Bcl2 expression	[51]
Peppermint	*Mentha piperita*	LNCaP	Inhibition of cell growth	[41]
Potato tree	*Salanum macranthum*	PC3	Inhibition of cell growth	[52]
Rose	*Rosa centifolia*	PC3	Inhibition of cell growth	[38]
Rosemary	*Rosmarinus officinalis*	LNCaP	Inhibition of cell growth	[53]
Sage	*Salvia aurea*	DU145	Inhibition of cell growth; induction of apoptosis; DNA fragmentation; increased LDH, caspase activity, Bax/Bcl-2 ratio and ROS generation; and decreased GSH	[54]
	*Salvia Judaica*	DU145	Inhibition of cell growth; induction of apoptosis; DNA fragmentation; increased LDH, caspase activity, Bax/Bcl-2 ratio and ROS generation; and decreased GSH	[54]
	*Salvia viscosa*	DU145	Inhibition of cell growth; induction of apoptosis; DNA fragmentation; increased LDH, caspase activity, Bax/Bcl-2 ratio and ROS generation; and decreased GSH	[54]
Spearmint	*Mentha spicata*	LNCaP	Inhibition of cell growth	[41]
		PC3	Inhibition of cell growth	[38]
Thyme	*Thymus vulgaris*	PC3	Inhibition of cell growth	[38]
Tsauri grass	*Cymbopogon giganteus*	PC3 and LNCaP	Inhibition of cell growth	[46]
Turmeric	*Curcuma longa*	PC3	Inhibition of cell growth	[52]
		LNCaP	Inhibition of cell growth	[55]
Wild mint	*Mentha* *arvensis*	LNCaP	Inhibition of cell growth	[41]
Wild spikenard	*Hyptis suaveolens*	LNCaP	Inhibition of cell growth and cell cycle arrest	[56]
	*Achillea wilhelmsii*	PC3	Inhibition of cell growth	[57]
	*Aloysia polystachya*	PC3	Inhibition of cell growth	[58]
	*Amomum tsao-ko*	PC3	Inhibition of cell growth	[59]
	*Anaxagorea brevipes*	PC3	Inhibition of cell growth	[60]
	*Annona sylvatica*	PC3	Inhibition of cell growth	[61]
	*Artemisia arborescens*	LNCaP and DU145	Inhibition of cell growth, DNA fragmentation and ROS generation	[62]
	*Bursera glabrifolia*	PC3	Inhibition of cell growth	[63]
	*Euodia ruticarpa*	PC3	Inhibition of cell growth	[64]
	*Guatteria elliptica*	PC3	Inhibition of cell growth	[65]
	*Hedychium spicatum*	PC3	Inhibition of cell growth, induction of apoptosis, cell cycle arrest, increased caspase activity, Bax/Bcl-2 ratio and ROS generation	[66]
	*Hedychium coccineum*, *Hedychium gardnerianum*, *Hedychium greenii* and *Hedychium. griffithianum*	PC3	Inhibition of cell growth	[67]
	*Hypericum hircinum*	PC3	Inhibition of cell growth	[68]
	*Iryanthera polyneura*	PC3	Inhibition of cell growth	[69]
	*Liquidambar orientalis*	PC3	Inhibition of cell growth	[70]
	*Symphyopappus itatiayensis*, *Myrciaria floribundus*, *Talauma ovata*, *Psidium cattleyanum*, *Nectandra megapotamica*	PC3	Inhibition of cell growth	[71]
	*Guatteria pogonopus*	PC-3M	Inhibition of cell growth	[72]
	*Perralderia coronopifolia*	PC3	Inhibition of cell growth	[73]
	*Pinus mugo*	DU145	Inhibition of cell growth and constitutive STAT3 activation; decreased expression of cyclin D1, Bcl-2, survivin, XIAP, Cox2 and IL-6; decrease in GSH levels and increase in ROS generation; induced caspase-3 and PARP cleavage; and inhibition of cell migration	[74]
	*Zataria Multiflora*	PC3	Inhibition of cell growth, induction of apoptosis, DNA fragmentation, cell cycle arrest, increased ROS generation and caspase activation, upregulation of Bax and downregulation of Bcl-2 expression	[75]

**Table 2:** Essential oils’ constituents investigated for their in vitro anti-prostate cancer potential and the major resultsreported.

Constituent	Plant	Cell Line	Effects	References
α-humulene	*Salvia* species	LNCaP	Inhibition of cell growth	[76]
α-pinene	Rosemary, lavender and others	PC3 and DU145	Inhibition of cell growth, induced apoptosis and cell cycle arrest	[77]
β-Caryophyllene oxide	Cinnamon, oregano, clove and black pepper	PC3	Inhibition of cell growth; induced apoptosis by inhibiting PI3K/AKT/mTOR/S6K1 signaling; reduced mitochondrial membrane potential; cytochrome c release; activating caspase-3; cleavage of PARP; ROS generation; downregulation of Bcl-2, Bcl-xL, survivin, IAP-1, IAP-2 and cyclin D1; upregulation of p53 and p21; and downregulation of COX-2 and VEGF	[78,79]
		DU145	Inhibition of cell growth and invasion and constitutive STAT3 activation	[80]
β-elemene	Curcuma and *Cymbopogon* species	PC3 and DU145	Inhibition of cell growth; induced apoptosis through cleaved caspase-3, caspase-9 and increasing PARP levels; and downregulated and Bcl-2	[81]
Carvacrol	Thyme, oregano and others	DU145	Inhibition of cell growth, induction of apoptosis, cell cycle arrest increased caspase-3 activation and ROS generation	[82]
		PC3	Inhibition of cell growth; migration and invasion; induction of apoptosis increased caspase activation and ROS generation; disruption of mitochondrial membrane potential; cell cycle arrest; upregulation of Bax and downregulation of Bcl-2 expression; decreased expression of Notch1, Jagged-1, cyclin D1 and CDK4; and increased expression of p21	[83]
		PC3 and DU145	Inhibition of cell growth, migration, and invasion; decreased TRPM7-like current and reduced MMP-2 protein expression and F-actin dynamics; alterations of PI3K/Akt and MEK/MAPK signaling pathways	[84,85]
Cinnamaldehyde	Cinnamon	PC3 and LNCaP	Inhibition of cell growth and proteasome activity; upregulated Hsp70 and downregulated VEGF and VEGFR expression	[86]
		Prostate CAF	Inhibition of cell growth, induction of apoptosis induction, cell cycle arrest, increased ROS generation and caspase activation and decreased GSH levels	[87]
		Prostate CAF	Relieves the immunosuppressive effects in a TLR4-dependent manner	[88]
Cinnamic acid	Cinnamon	PC3 and LNCaP	Inhibition of cell growth and proteasome activity; upregulated Hsp70 and downregulated VEGF and VEGFR expression	[86]
Citral	*Cymbopogon* species	PC3 and PC3M	Inhibition of cell growth, reduced clonogenic potential, induced morphological alterations, expulsion of lipid droplets, activation of AMPK protein expression, induction of apoptosis, DNA fragmentation, upregulation of Bax and downregulation of Bcl-2 expression	[89]
Eugenol	Cinnamon, clove and others	DU145	Inhibition of cell growth	[90]
		PC3 and LNCaP	Inhibition of cell growth and proteasome activity; upregulated Hsp70 and downregulated VEGF and VEGFR expression	[86]
Geraniol	*Cymbopogon* species	PC3	Inhibition of cell growth; increased LDH and caspase-3 activity; induced mitochondrial membrane depolarization and cell cycle arrest at the G1 phase; reduced expressions of cyclin A, B, D and E, CDK1 and CDK4, and Bcl-2 and Bcl-w; elevated expressions of p21 and p27 and Bax and BNIP3	[91]
			Inhibition of cell growth, induced autophagy and inhibited AKT-mTOR signaling	[92]
			Downregulated the transcription factor E2F8	[93]
Limonene	*Citrus* and *Mentha* species	DU145	Inhibition of cell growth, induction of apoptosis, ROS generation, DNA fragmentation, caspase-3 and caspase-9 cleavage, upregulation of p21, p53 and Bad, downregulation of Bcl-xL and cleavage of PARP protein	[94]
Linalool	Lavender, *Salvia* species and others	PC3 and DU145	Inhibition of cell growth and migration, induction of apoptosis and cell cycle arrest	[44]
		22Rvl	Inhibition of cell growth; induction of apoptosis; cell cycle arrest; increased expression of Bax, Bcl-2, p53, DR4, DR5; and cleaved caspases	[95]
Linalyl acetate	Lavender, *Salvia* species and others	PC3 and DU145	Inhibition of cell growth and migration, induction of apoptosis and cell cycle arrest	[44]
Menthol	*Mentha* species	PC3, LNCaP and DU145	Inhibition of cell growth	[96]
		PC3 and LNCaP	Inhibition of cell growth	[97]
		LNCaP	Inhibition of cell growth	[98]
		DU145	Inhibition of cell growth and migration and cell cycle arrest	[99]
Thymol	Thyme	PC3 and DU145	Inhibition of cell growth and induction of apoptosis	[100]
